# Supplementary material for: Emergent chirality in a polar meron to skyrmion phase transition
Source: Nat Commun. 2023 Mar 13;14:1355. doi: 10.1038/s41467-023-36950-x (PMC10008831; doi:10.1038/s41467-023-36950-x)
Supplement: Supplementary file 1 — Supplementary Information [file 41467_2023_36950_MOESM1_ESM.pdf]

Supplementary Information for
Emergent chirality in a polar meron to skyrmion phase transition.

Yu-Tsun Shao[1,2], Sujit Das[3,4], Zijian Hong[5,6], Ruijuan Xu[7,8,9], Swathi Chandrika[1], Fernando Gómez-Ortiz[10], Pablo García-Fernández[10], Long-Qing Chen[5], Harold Y. Hwang[7,8], Javier Junquera[10], Lane W. Martin[4,11], Ramamoorthy Ramesh[4,11,12] & David A. Muller[1,13].

Corresponding author: David A. Muller
Email: david.a.muller@cornell.edu

This Word file includes:

Supplementary text
Figures S1 to S14
SI References

Supplementary Information Text

Supplementary notes on topology of a polar meron. From a topology perspective, the most important difference between a skyrmion and a meron is the evolution of out-of-plane component of the polarization from the core to periphery. In a skyrmion, the out-of-plane component changes from a direction at the core to the opposite direction at the periphery. Whereas in a meron, the out-of-plane component changes from a direction at the core to be totally confined in-plane at the periphery. As an example, a meron and a skyrmion can have the same in-plane texture and vary only by their out-of-plane components. Take the merons shown in Fig.4 for an example, the core region (with an out-of-plane component of the polarization) is not fully surrounded by a region with the antiparallel out-of-plane polarization. Instead, now each region with an out-of-plane component of the polarization is confined within a region with in-plane polarization at the periphery.

Second, the regions with an opposite component of the out-of-plane polarization *always appear at the center of an anti-vortex* (see the red crosses in Figs. 4B, 4D, 4F, and Fig. S9B). Therefore, the presence of an out-of-plane component of the polarization does not penalize the appearance of the meronic phase. Just the opposite, it enhances it since the regions surrounding the up and down polarizations contribute with the same skyrmion number of -1/2, as shown in Fig. 1.

Sample preparation using RHEED-assisted pulsed-laser deposition. The epitaxial lift off sacrificial layer of 16 nm $Sr_2CaAl_2O_6$ with a 2.4 nm $SrTiO_3$ capping layer was synthesized on single-crystalline $SrTiO_3$ (001) substrates via reflection high-energy electron diffraction (RHEED)–assisted pulsed laser deposition. The growth of the $Sr_2CaAl_2O_6$ layer was carried out in a dynamic argon pressure of 4×10^{-6} Torr, at a growth temperature of 710 °C, a laser fluence of 1.35 J/cm^2, and a repetition rate of 1 Hz, using a 4.8 mm^2 imaged laser spot. The growth of the $SrTiO_3$ layer was conducted in dynamic oxygen pressure of 4×10^{-6} torr, at a growth temperature of 710 °C, a laser fluence of 0.9 J/cm^2, and a repetition rate of 1 Hz, using a 3.0 mm^2 imaged laser spot. The heterostructure was then cooled down to room temperature at the growth pressure. Subsequent to this, n-$SrTiO_3$/n-$PbTiO_3$/n-$SrTiO_3$ trilayers (n- is the number of monolayers; n=16) and $[(PbTiO_3)_{16}/(SrTiO_3)_{16}]_8$ superlattices were synthesized *ex-situ* on this template via RHEED-assisted pulsed-laser deposition (KrF laser). The $PbTiO_3$ and the $SrTiO_3$ were grown at 610 °C in 100 mTorr oxygen pressure. For all materials, the laser fluence was 1.5 J/cm^2 with a repetition rate of 10 Hz. RHEED was used during the deposition to ensure the maintenance of a layer-by-layer growth mode for both the $PbTiO_3$ and $SrTiO_3$. The specular RHEED spot was used to monitor the RHEED oscillations. After deposition, the heterostructures were annealed for 10 minutes in 50 Torr oxygen pressure to promote full oxidation and then cooled down to room temperature at that oxygen pressure.

Membrane lift-off and transfer. The heterostructure was first spin-coated with a polymer support of 500 nm thick polymethyl methacrylate (PMMA) film and placed in deionized water at room temperature until the sacrificial $Sr_2CaAl_2O_6$ layer was fully dissolved. The PMMA coated film was then released from the substrate and transferred onto the TEM gird (NH050D2, Norcada Inc.). Finally, the PMMA layer was dissolved and removed from the membrane in acetone, as schematically shown in Fig. S12. The membrane sample surface was further cleaned in ozone at 180 °C for 10 mins.

X-ray structural analysis. In order to obtain a comprehensive picture of the crystal structure of superlattices, as well as information on the in-plane and out-of-plane ordering, was carried out using a Panalytical X'Pert Pro X-ray Diffraction (XRD) diffractometer with Cu-K_α radiation (λ = 1.5405 Å). The high crystalline quality of the films, the smooth nature of the interfaces and the

skyrmion ordering, was confirmed from reciprocal space mapping of the as-grown and lifted-off superlattice.

Phase-field calculations. Phase-field simulations were performed to study equilibrium polar structures of $(PTO)_{16}/(STO)_{16}$ superlattice under different temperatures and strain conditions. The spontaneous polarization vector $\vec{P}$ is used as the primary order parameter. The temporal evolution of $\vec{P}$ is governed by the time dependent Ginsburg-Landau equation, i.e.,

$$\frac{\partial P_i}{\partial t} = -L\frac{\delta F}{\delta P_i} \quad (i = 1{,}3)$$

Where L is the kinetic coefficient, t is the evolution time. The total free energy F can be obtained by integrating the contributions from the mechanical, electrical, Landau chemical and polar gradient energies,

$$F = \int (f_{Elastic} + f_{Electric} + f_{Landau} + f_{Gradient})dV$$

Detailed expressions of the energy terms, materials parameters as well as the numerical simulation procedure is described in the published literature[1–5]. The simulation system is discretized into a three-dimensional grid of 200Δx×200Δy×350Δz, with Δx=Δy=Δz=0.4 nm. A periodic boundary condition is used along the in-plane dimensions, while a superposition method is applied in the out-of-plane direction[6]. In the out-of-plane direction, the thickness of the substrate, film and air are set as 30Δz, 288Δz and 32Δz, respectively; while the film is comprised of periodic stacking of 16Δz of PTO layers and 16Δz of STO layers. A closed-circuit, electric boundary condition is assumed where the electric potential is fixed to zero at the top and bottom of the film surface[2]. When a thin film boundary condition is applied where the stress on the top of the thin film is zero, and the displacement at the bottom of the substrate sufficiently far away from the film/substrate interface is set to zero[3]. An iteration-perturbation method is adopted to account for the inhomogeneity in the elastic constants of PTO and STO[7]. To determine the local strain state, the reference pseudocubic lattice constants for STO (PTO) at 223K, 300 K and 373 K are set as 3.901 Å (3.953 Å), 3.905 Å (3.957 Å) and 3.909 Å (3.961 Å), respectively. The average effective substrate lattice constants are taken from experimental measurements, which are set as a=3.875 Å, b=3.885 Å for 223 K, a=b=3.905 Å for 300 K and a=b=3.899 Å for 373 K. The large compressive strains are due to local bending when the lift-off membrane is heated/cooled down. A background dielectric constant of 40 is used[8,9]. Random noise with a magnitude of 0.0001 C/m^2 is added to the system as the initial polarization distribution of the system. The polar structures are shown in Figs. 2G-I & S8. At 223K, due to the anisotropic bending of the membrane during cooling, elongated skyrmion stripes, or bimerons, are formed along X-axis. Meanwhile, at room temperature, a disordered skyrmion lattice is observed, consistent with a previous report[10]. When the system is further heated up to 373K, a locally ordered square skyrmion lattice is observed. The formation of this locally ordered square lattice is attributed to the large local compressive strain that generated due to the bending of the membrane during heating. Interestingly, some dislocation-like features are also observed, which could locally disturb the ordered structure, consistent with experimental observations.

STEM. The plan-view samples of the $[(PbTiO_3)_{16}/(SrTiO_3)_{16}]_8$ superlattices were lifted-off and transferred to TEM grids. Cross-sectional TEM specimens were prepared on the same plan-view samples, using a FEI Strata 400 focused ion beam (FIB) with a final milling step of 2 keV to reduce damage. The initial sample surface was protected from ion-beam damage by depositing carbon and platinum layers prior to milling. The cross-sectional TEM specimen has a thickness of ~25 nm as determined by CBED analysis. HAADF-STEM images were recorded by using a Cs-corrected FEI Titan operated at 300 keV, with beam semi-convergence angle of 21.4 mrad and beam current of 30 pA.

SCBED for polarization mapping. We performed scanning convergent beam electron diffraction (SCBED) experiments using an electron microscopy pixel array detector (EMPAD), where the 2D electron diffraction pattern was recorded over a 2D grid of real space probe positions, resulting in 4D datasets. Experimental data was acquired using a FEI Titan operated at 300 keV with 15 pA beam current, 2.45 mrad semi-convergence angle, having a probe of ~8 Å FWHM (full-width at half-maximum). A double-tilt liquid-nitrogen-cooled Gatan specimen holder was used for temperature-dependent studies ranging from 95 K to 373 K. The CBED patterns were captured by the EMPAD with exposure time set to 1 ms per frame, for which a 256 × 256 scan can be recorded in under 2 minutes. Due to dynamical diffraction effects, the charge redistribution associated with polarization leads to the breakdown of Friedel's law. From the collected CBED patterns, the polarization direction in the plan-view samples is reconstructed by calculating the center-of-mass in polarity sensitive Kikuchi bands along the cubic directions. We employ the Kikuchi bands as a more robust means to extract polarity information against internal crystal mis-tilts, which often occurs in ferroic oxides due to disinclination strain. In addition, due to electron channeling effects, the polarity information obtained from Kikuchi bands at this experimental condition is mostly arising from the topmost $PbTiO_3$ layer, which overcomes the problem of overlapping signals from $PbTiO_3$ multilayers projected along the plan-view geometry (Fig. S2). For $SrTiO_3/PbTiO_3/SrTiO_3$ trilayer samples, Friedel pairs of Bragg reflections were used, such as (300)/($\bar{3}$00) and (030)/(0$\bar{3}$0) for x and y components of polarization, respectively (Fig. S3). By matching with dynamical diffraction simulations (Fig. S5), we can unambiguously determine the polarization directions in real space. We note that due to different channeling conditions from different probe semi-convergence angles of 21.4 mrad and 2.45 mrad, for plan-view imaging, atomic-resolution STEM and 4D-STEM map the Néel and Bloch components of a polar skyrmion, respectively (Fig. S4). For clarity of display, the polarization maps obtained using the Kikuchi bands method were first subtracted a low frequency background, then smoothed using a Gaussian filter of 3 pixels. The details in filtering are shown in Fig. S13.

SCBED for chirality determination. In order to excite the higher order Laue zone (HOLZ) reflections, the plan-view samples were deliberately tilted ~6.2° away from the [001] zone axis, along one of the mirror planes (Fig. S10A). We then perform SCBED experiments exactly at this diffraction condition at various temperatures. Within a SCBED dataset, we carefully selected local regions with minimal tilt and thickness variations. By using dynamical diffraction simulations as the reference, the chirality can thus be determined by comparing the intensity asymmetry of Bijvoet pairs, such as (671)/($\bar{6}$71) and (771)/($\bar{7}$71).

SCBED for strain analysis. We perform exit wave power cepstrum (EWPC) analysis on SCBED datasets to look at changes in lattice parameters. The EWPC works by a discrete Fourier transform of the logarithm of a CBED pattern, resulting units in real-space. Figure S7B shows an EWPC pattern, in which the peaks correspond to projected Pb-Pb inter-atomic distances. Thus, the change in mean projected, in-plane lattice parameters can be measured by comparing the peak distances in EWPC patterns. Sub-picometer precision can be achieved by sub-pixel peaking fitting using the algorithm described previously[11]. For the sake of self-consistency, SCBED datasets for temperature-dependent strain analysis were acquired using the exact same TEM optics, within one experimental session. To produce the histogram in Fig. S7, each diffraction pattern was probed from a 1nm diameter spot, and strain from that diffraction pattern was calculated. We recorded 128x128=16,384 diffraction patterns from a 100×100 nm^2 region. The histogram was based on 5 of such regions, which is >80,000 diffraction patterns. Consequently, what we have is a histogram of local in-plane lattice parameter variations over an area of 500 nm^2, rather than an averaged lattice parameter. In other words, SCBED is probing the distributions of local textures rather than the ensemble average.

Dynamical diffraction simulation. The CBED simulations were carried out using the μSTEM software[12], with neutral atomic scattering factors of Waasmaier & Kirfel[13]. The atomic coordinates were taken from results of 2nd-principles simulations for a right-handed polar skyrmion. 25×25 diffraction patterns with a 3.2-Å scan step size were simulated at 300-keV beam energy and with 2.45-mrad semi-convergence angle. To simulate Kikuchi bands, thermal diffuse scattering effect was included with the frozen-phonon approximation.

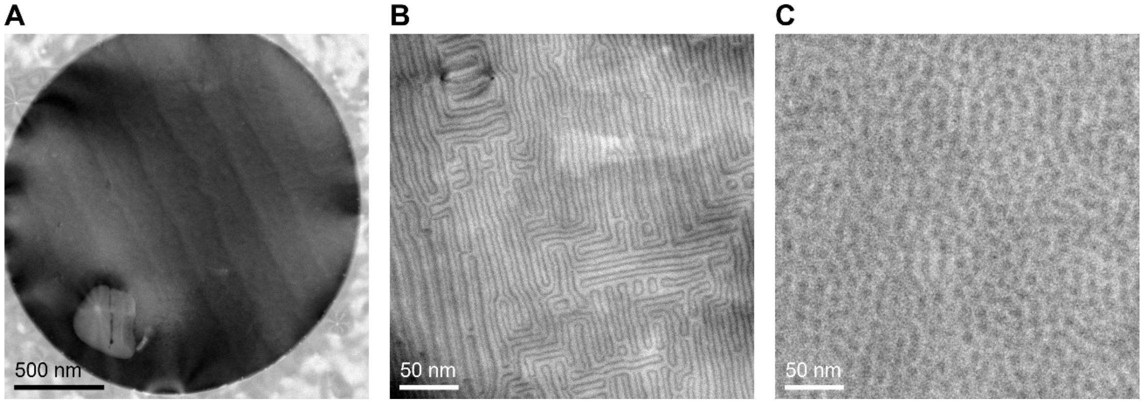

Fig. S1. Lifted-off, freestanding membrane of $(PbTiO_3)_{16}/(SrTiO_3)_{16}$ heterostructures for plane-view TEM. (**A**) Low-magnification STEM image showing the oxide membrane suspended over a ~2 μm SiN_x hole of the TEM grid. Exemplar STEM images of (**B**) a trilayer and (**C**) a superlattice acquired from freestanding regions and at room temperature.

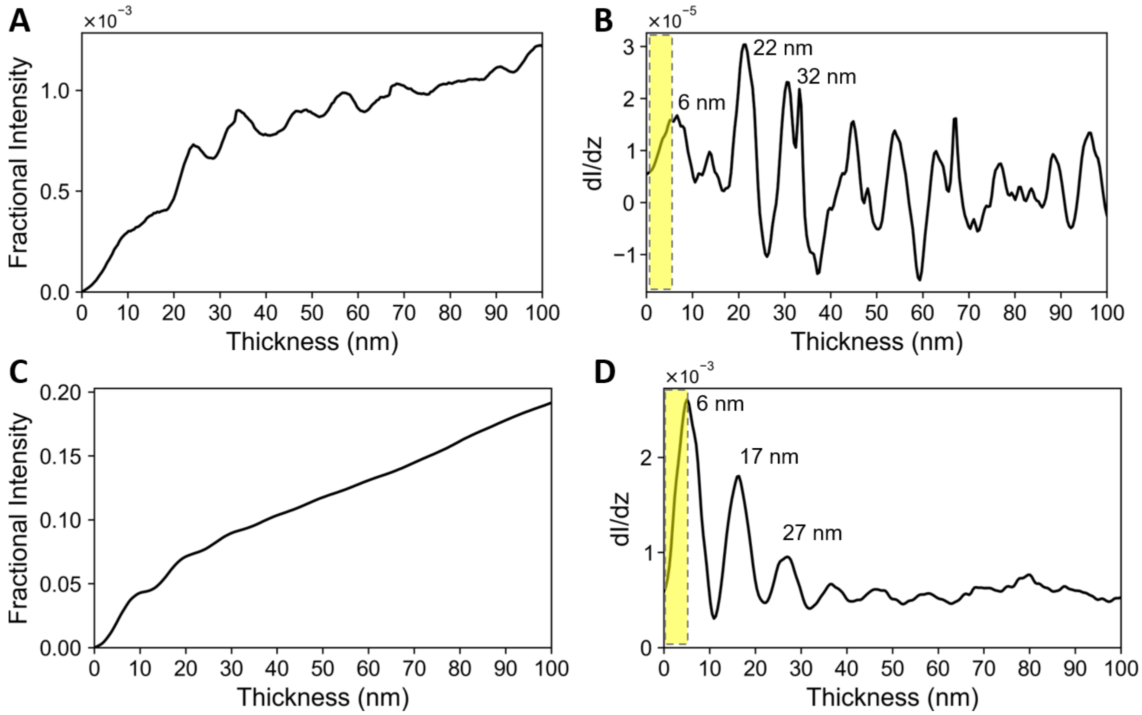

Fig. S2. Electron beam channeling along a column of Sr atoms in $SrTiO_3$. The intensity $\boldsymbol{I(z)}$ of (**A**) (300) Bragg reflection and (**C**) high-angle ADF signal (40-100 mrad) for a 300-keV electron beam centered on the Sr-site as a function of depth *z* into the crystal. The focused probe with semi-convergence angle of 2.45 mrad is similar to that used in the EMPAD experiments to separate diffraction disks. (**B**) The derivative of the (300) diffracted intensity, showing several peaks of signal are generated at 6 nm, 22 nm, 32 nm into the $SrTiO_3$, corresponding to the points where 1st, 2nd, and 3rd $PbTiO_3$ layers would begin in the multilayer structure. (**D**) From derivative $\boldsymbol{dI/dz}$, the signal is channeling most efficiently at 6 nm. The yellow box shows the thickness of 16 unit cells of $SrTiO_3$. By changing the collection angle, the Kikuchi bands are more suitable than (300) reflections for retrieving polarization information from a single (top) $PbTiO_3$ layer in the repeated cell in the 16×16 multilayer structure.

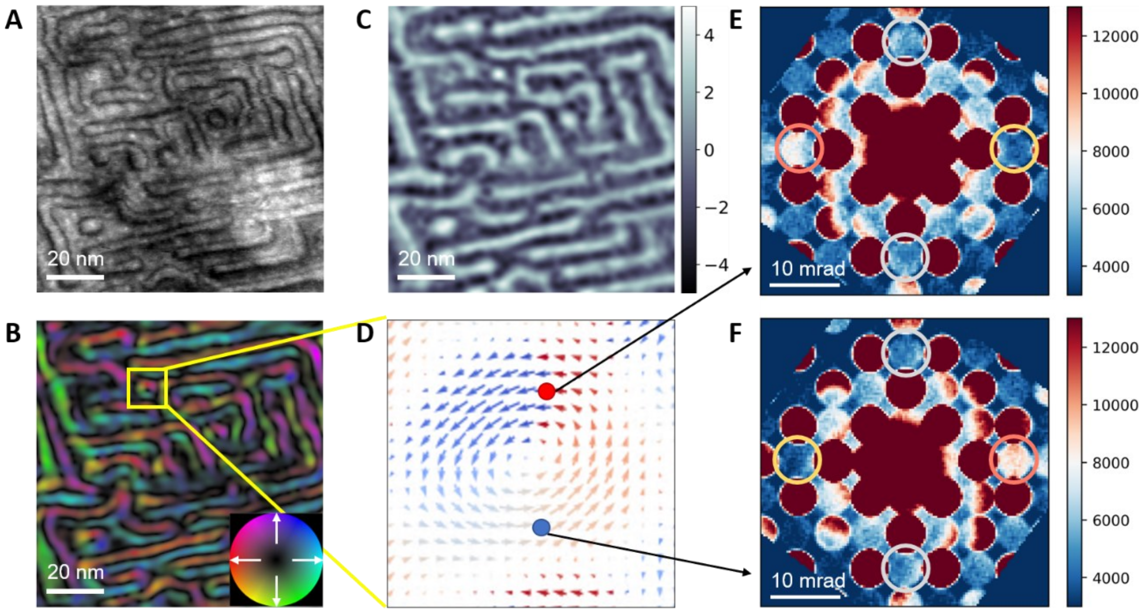

Fig. S3. Imaging internal Bloch components of polar-skyrmions in the $(SrTiO_3)_{16}/(PbTiO_3)_{16}/(SrTiO_3)_{16}$ trilayer. (**A**) <100> dark field image and (**B**) polarization map reconstructed from the SCBED dataset showing the in-plane Bloch components of polar-skyrmions and labyrinth phase. The color map in (B) represents the in-plane polarization direction at each point. (**C**) Curl of the in-plane polarization. (**D**) Magnified skyrmion from the yellow box in (B). Ferroelectric polarization direction can be determined by observing the difference of diffracted intensities of Friedel pairs, $\boldsymbol{I}_{\vec{G}}$ and $\boldsymbol{I}_{-\vec{G}}$. Representative CBED patterns taken from (**E**) top and (**F**) bottom of a skyrmion, where the polarity-sensitive <300> Bragg reflections are selected for determining polarization, as marked by pink and yellow circles.

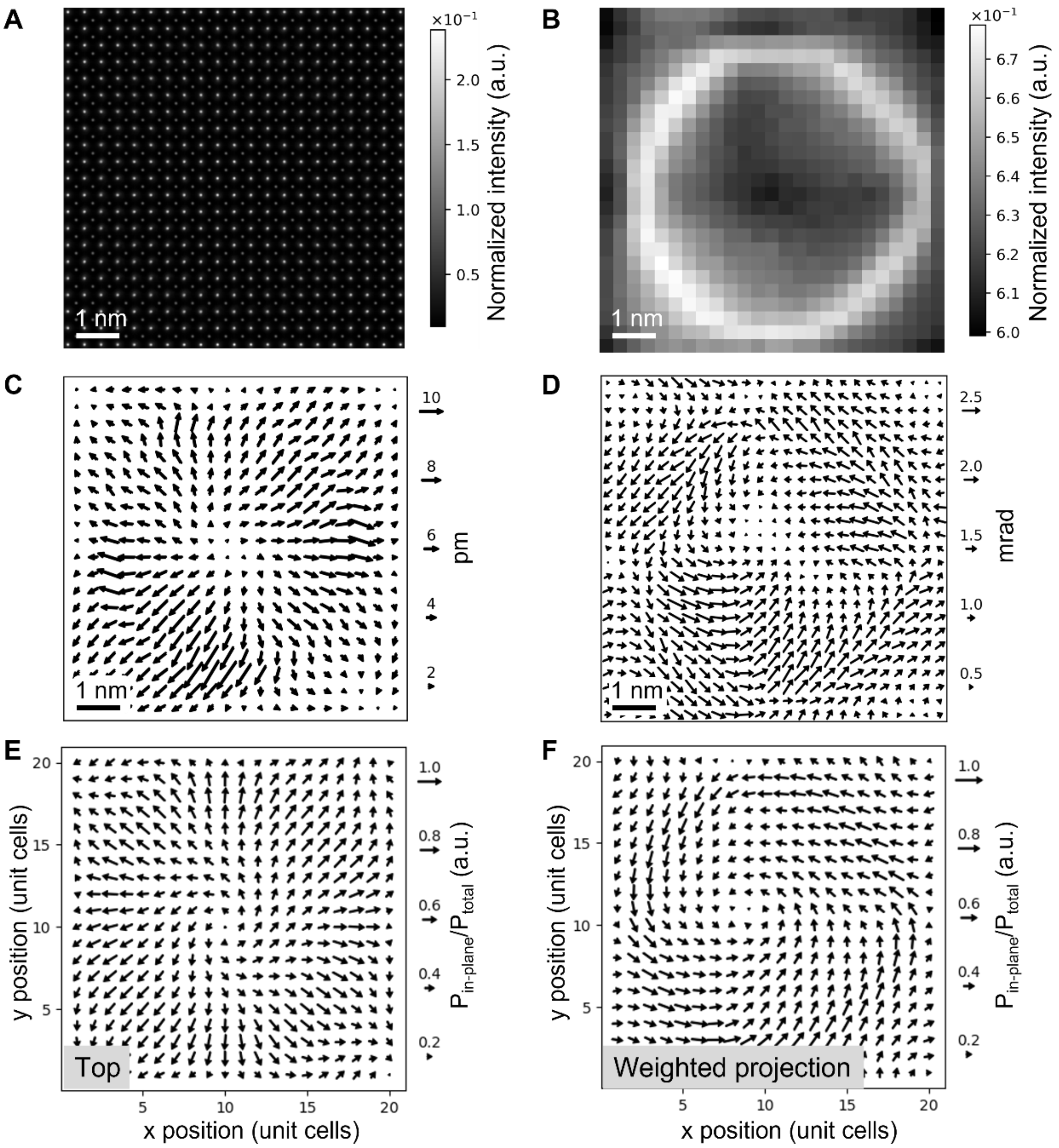

Fig. S4. Plan-view polarization mapping of a polar skyrmion using atomic-resolution HAADF-STEM versus 4D-STEM Kikuchi bands. Simulated images for both (**A**) atomic resolution HAADF-STEM and (**B**) ADF-STEM image generated from the multislice-calculated 4D-STEM dataset. (**C**) Ti-displacement vector map obtained from (A), and (D) polarity map obtained from Kikuchi bands in the simulated 4D-STEM dataset in (B). The in-plane polarization component obtained from second-principles calculations of the skyrmion structure showing (**E**) hedgehog-like structure at the top and (**F**) Bloch vortex from weighted projection with more on the central plane in $PbTiO_3$. The HAADF-STEM and 4D-STEM datasets are simulated using a 300 keV electron, with probe semi-convergence angles of 21.4 mrad and 2.45 mrad, respectively. Due to difference in channeling conditions (the more convergent HAADF-STEM dechannels much closer to the entrance surface), HAADF-STEM and 4D-STEM are sensitive to Néel and Bloch components of the polar skyrmion, respectively.

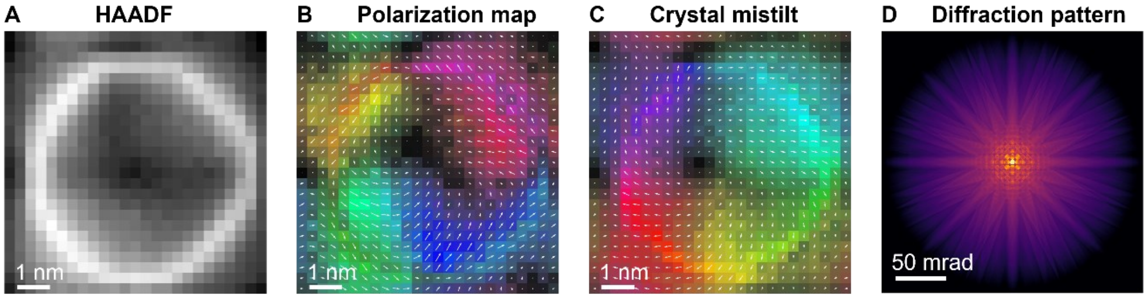

Fig. S5. Decoupling polarization and crystal mistilts in a polar skyrmion. The 4D-STEM/SCBED dataset of a polar skyrmion simulated using coordinates from second-principles calculations, with a 300-keV electron beam and a focused probe semi-convergence angle of 2.45 mrad. (**A**) High angle annular dark field (HAADF) image generated by integrating an angular range of 50-130 mrad. (**B**) Polarization map obtained using the Kikuchi band method. (**C**) Effective crystal mistilts information can be extracted from the intensity redistribution in the (000) disk. (**D**) Averaged CBED pattern shown in logarithmic scale. Note the maps of polarization and crystal mistilts can be decoupled using different parts in the CBED patterns.

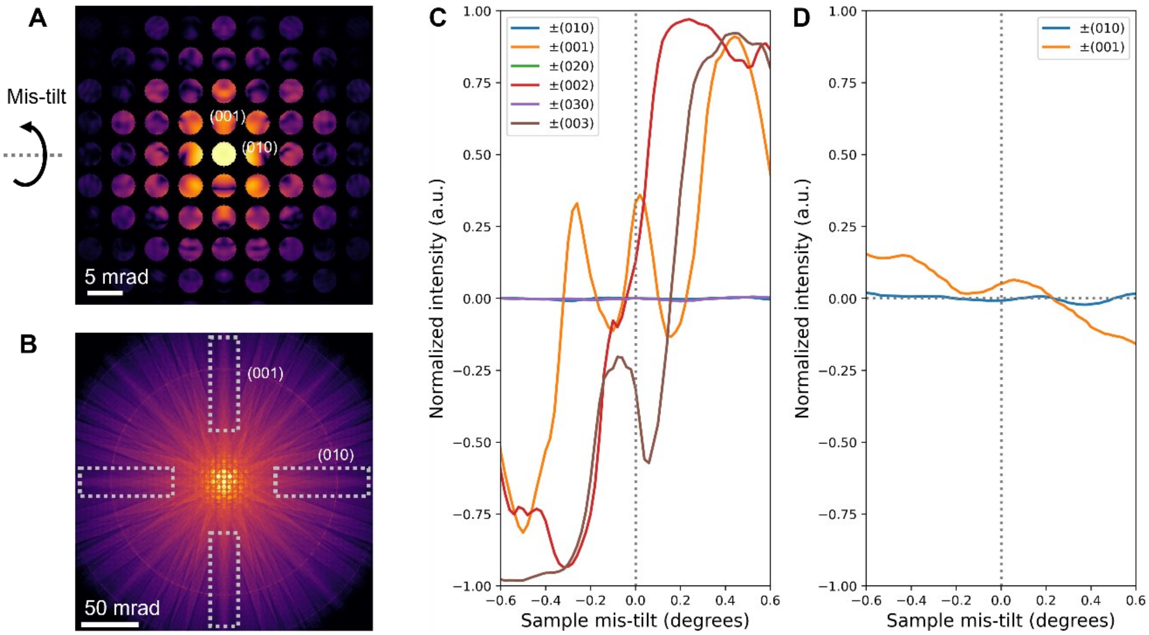

Fig. S6. **Diffraction effects and mis-tilt artifacts on the polarity determination of $PbTiO_3$.** Due to dynamical diffraction effects, the polarity-sensitive (A) Bragg reflections (e.g., ±(001), ±(002), and ±(003)) and (B) Kikuchi bands (e.g., ±(001)) along the polar axis exhibit intensity asymmetry. However, this intensity asymmetry can be largely affected by sample mis-tilt artifacts. Here, we simulate the CBED patterns of $PbTiO_3$ along $[100]_{pc}$ zone-axis with systematic crystal mis-tilt angles. (C) The intensity asymmetry in Bragg reflections can have contrast reversal with mis-tilts as small as 0.04º, while the Kikuchi bands in (D) can tolerate mis-tilt angles up to 0.23º. The diffraction patterns were simulated using 300 keV electrons with probe semi-convergence angle of 2 mrad, and $PbTiO_3$ thickness of 101 nm as consistent with the superlattice membrane. The intensity asymmetry is normalized as $\boldsymbol{I_{asymmetry}} = {(\boldsymbol{I_{hkl}} - \boldsymbol{I_{\bar{h}\bar{k}\bar{l}}})}/{(\boldsymbol{I_{hkl}} + \boldsymbol{I_{\bar{h}\bar{k}\bar{l}}})}$.

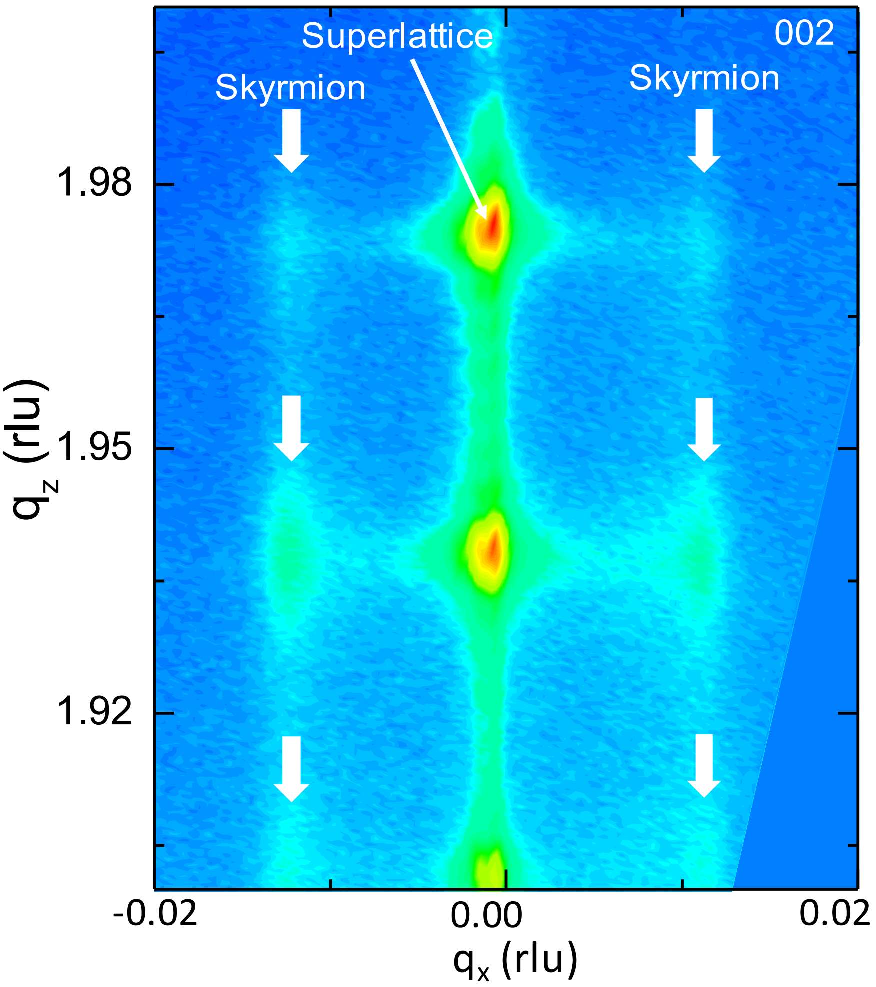

Fig. S7. Reciprocal space mapping of the $[(PbTiO_3)_{16}/(SrTiO_3)_{16}]_8$ superlattice lifted-off membrane. The white arrows indicate the satellite peaks associated with polar skyrmion modulation in the superlattice.

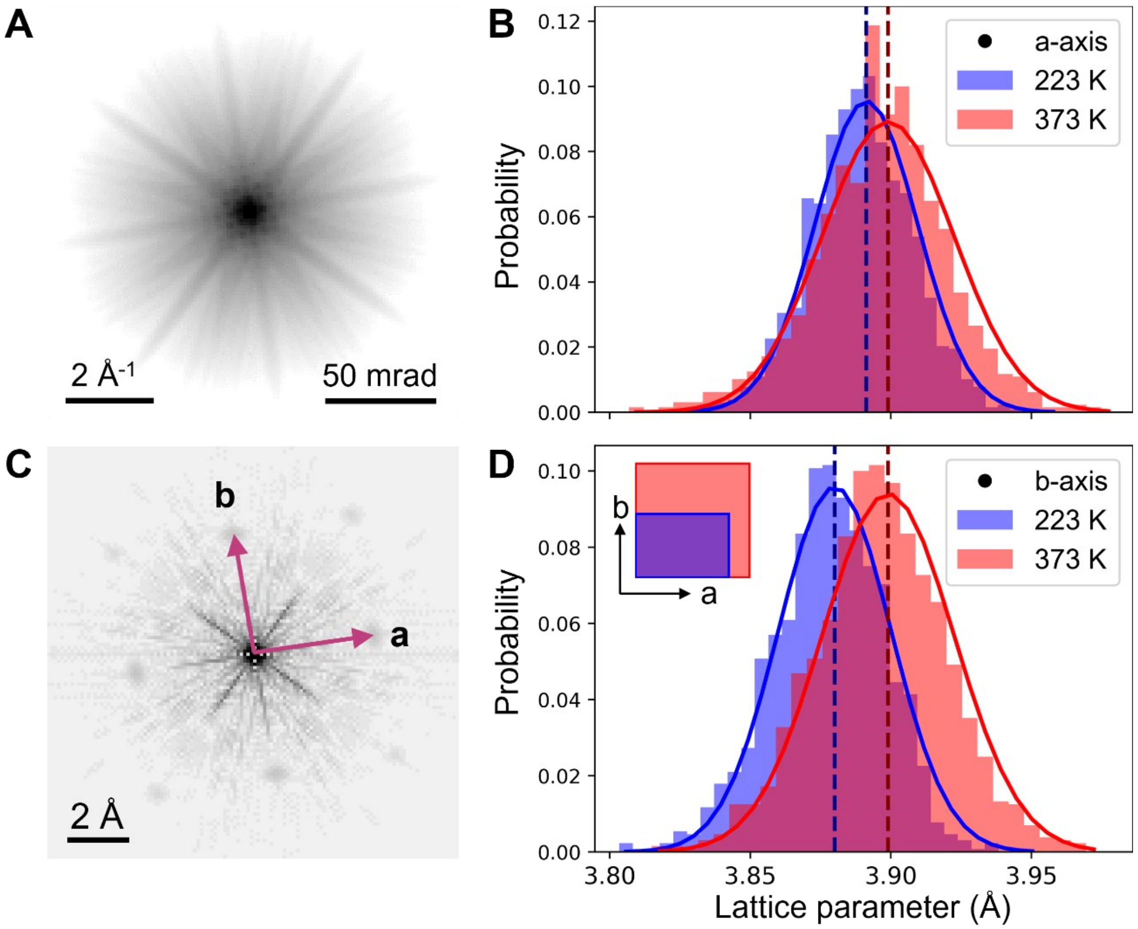

Fig. S8. Temperature-dependent strain analysis of $[(PbTiO_3)_{16}/(SrTiO_3)_{16}]_8$ superlattice. (**A**) Representative CBED pattern shown in logarithmic scale. (**C**) Exit wave power cepstrum (EWPC) transformation of (A) shows peaks corresponding to projected Pb-Pb inter-atomic distances in real-space. Spots that correspond to the length of projected distances along (100) and (010) are selected for tracking changes in lattice parameters *a* and *b*, respectively. Histograms of lattice parameters along (**B**) *a*- and (**D**) *b*-axis, at temperatures of 223 K (blue) and 373 K (red), over regions of ~500 nm^2 (>80,000 CBED patterns). The relative change in mean of *a*- and *b*-axis are ~0.2% and ~0.5%, respectively. Inset in (**D**) is an exaggerated cartoon of projected lattice parameters, indicating the film is more rectangular at 223 K than at 373 K.

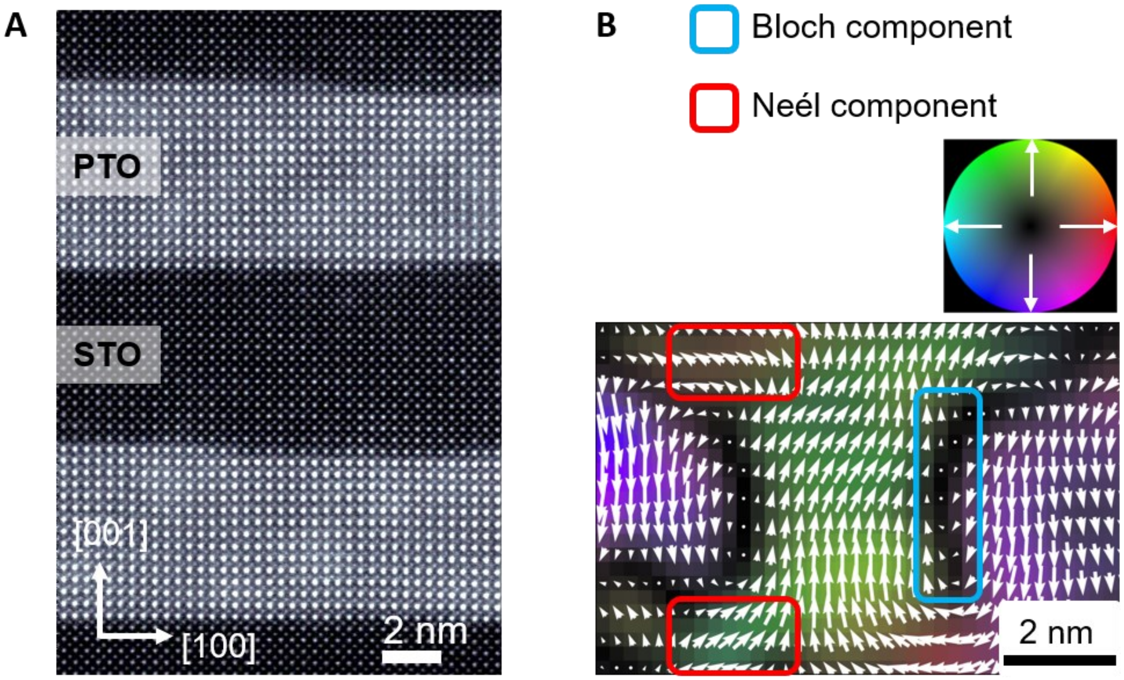

Fig. S9. Out-of-plane polarization configurations of polar skyrmions. (**A**) Cross-sectional HAADF-STEM image of the ($PbTiO_3$)/($SrTiO_3$) superlattice. (**B**) Polarization configuration reconstructed from the SCBED dataset, where we can access the cross-section of both Neél (red box) and Bloch (blue box) components of polar skyrmions. The out-of-plane polarizations are separated by in-plane Bloch chiral domain walls (dark regions).

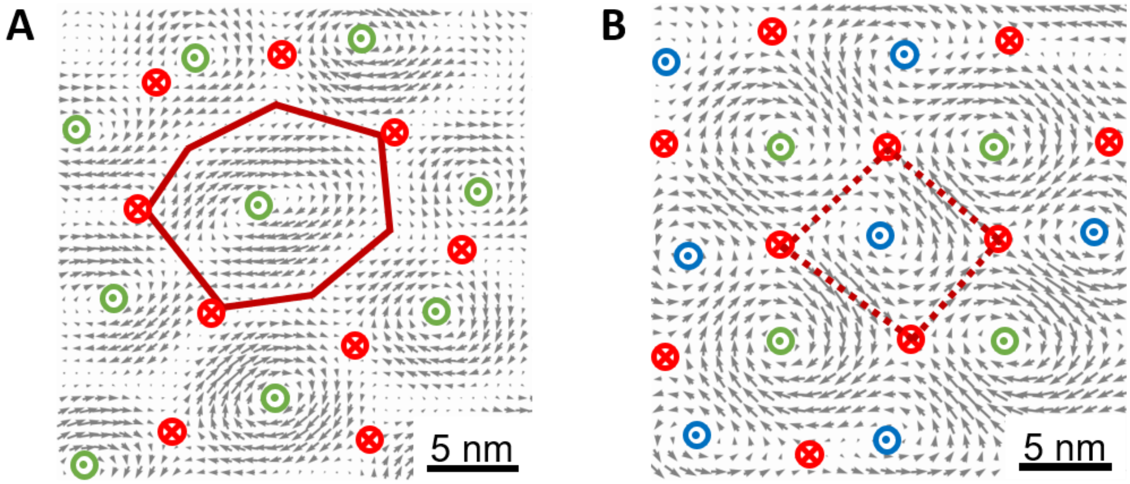

Fig. S10. Difference in topology between a skyrmion and a meron. Details of polarization for (**A**) a skyrmion and (**B**) a meron, where vortices (clockwise: green, counter-clockwise: blue) and anti-vortices (red) are labeled. The dots in circles represent out-of-plane polarization pointing towards out of the page, while the cross points into the page. The red solid lines in (**A**) form a closed loop of P_{op} (into the page) surrounding the core of the skyrmion with an antiparallel P_{op} (out of the page). In contrast, the P_{op} cannot form a closed loop around the core of the meron shown in (**B**), as indicated by dashed red lines.

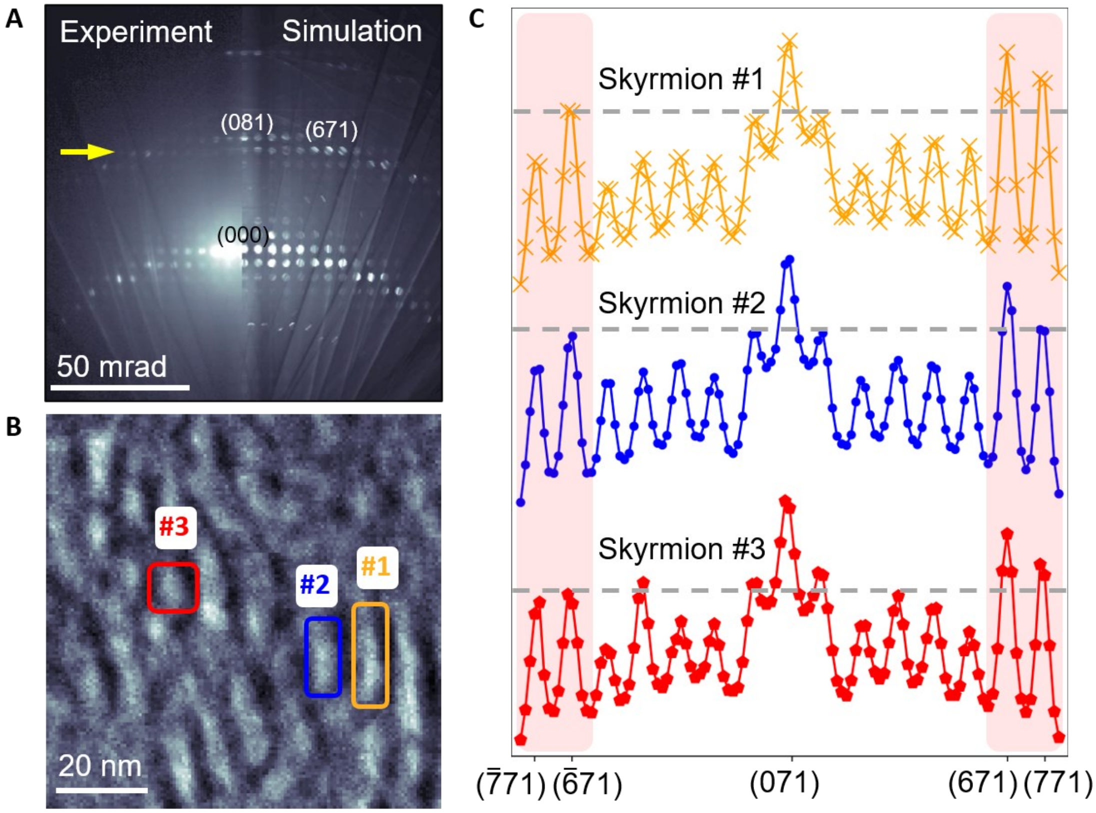

Fig. S11. Handedness determination of the chiral polar skyrmions at room temperature. (**A**) Representative experimental (left) and simulated (right) CBED pattern at incidence of ~6.2° away from [001] zone axis, tilted along one of the mirror planes. (**B**) Virtual dark field image reconstructed from the SCBED dataset using (081) reflection. (**C**) Intensity line profiles taken from a row of HOLZ reflections from selected regions labeled in (**B**), displaying the intensity difference between two pairs of reflections: (671) and $(\bar{6}71)$, (771) and $(\bar{7}71)$. The yellow arrow in (**A**) indicates the region where line profiles were drawn from.

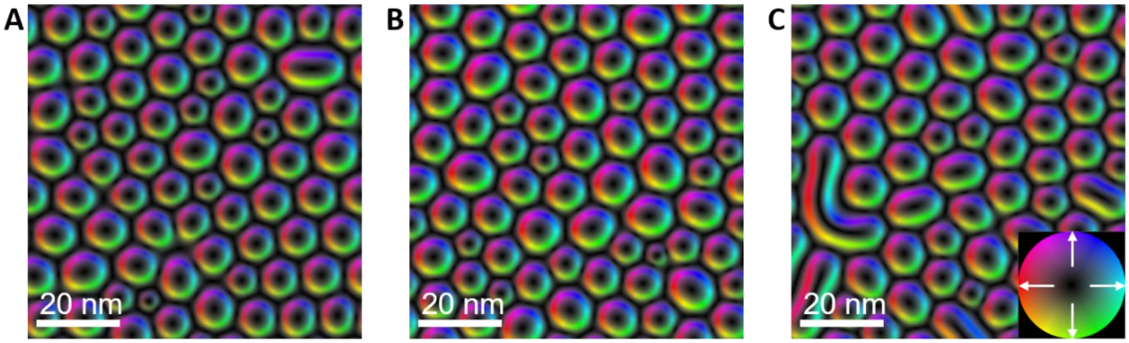

Fig. S12. Phase field simulations of polar skyrmion textures at different temperatures and under the same strain state. The strain is imposed on the $[(PbTiO_3)_{16}/(SrTiO_3)_{16}]_8$ superlattice by varying the substrate having in-plane lattice parameters (*a*,*b*) with *a*=*b*=3.905 Å, and at temperatures of (**A**) 223 K; (**B**) 298 K; and (**C**) 373 K. The color wheel hue (saturation) corresponds to the direction (magnitude) of the in-plane component of the ferroelectric polarization.

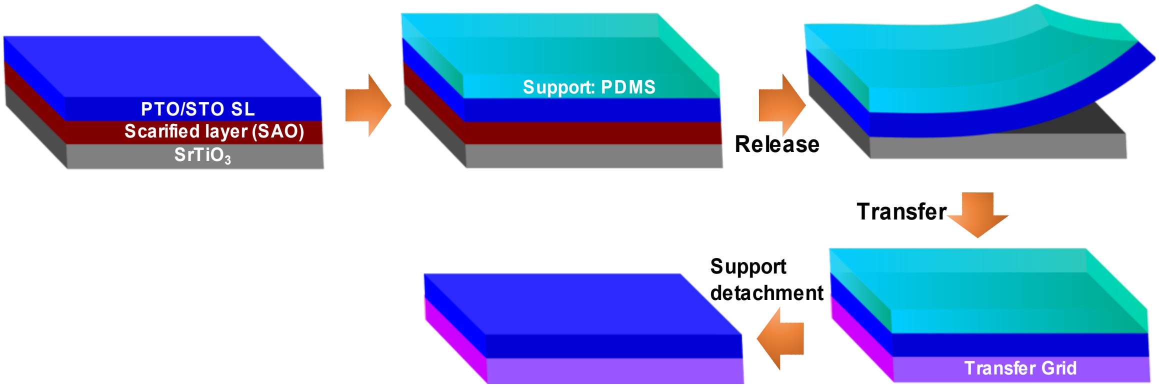

Fig. S13. Growth and transfer of $PbTiO_3$/$SrTiO_3$ (PTO/STO) superlattice. The Schematic of a superlattice with a $Sr_2CaAl_2O_6$ (SAO) sacrificial buffer layer. The sacrificial SAO layer is dissolved in water to release the top oxide films with the mechanical support of PMMA. The freestanding film is then transferred onto the desired substrate or TEM grid.

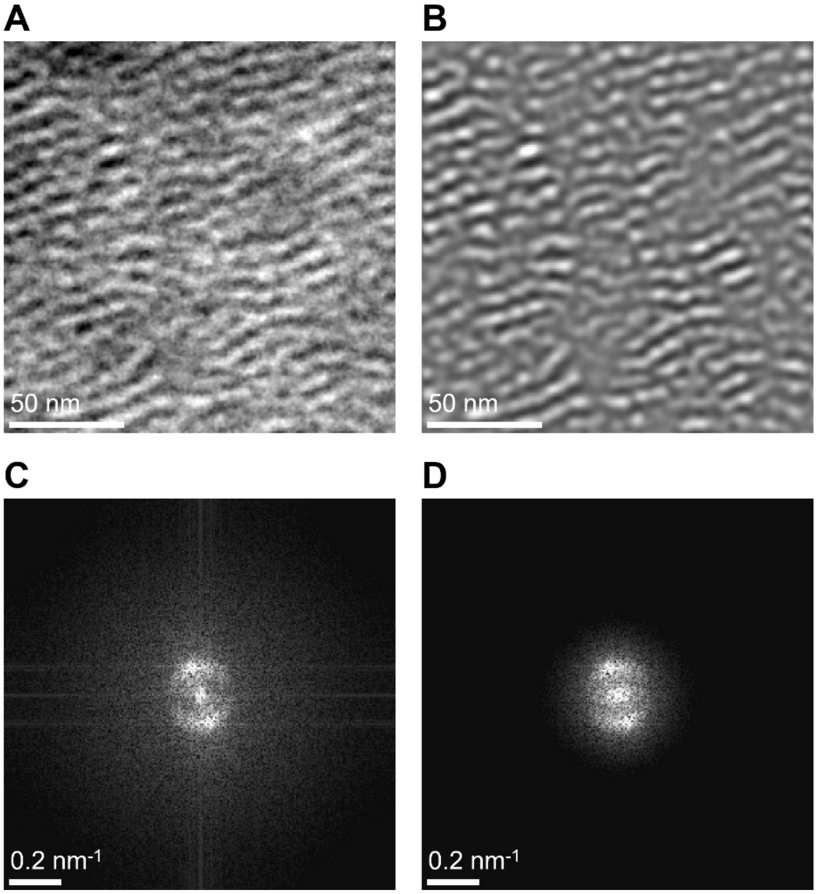

Fig. S14. Image processing details of the polarization components image. For clarity of display, the polarization map components reconstructed from the SCBED dataset were first subtracted off the low-frequency background with the rolling ball algorithm, then applied a Gaussian filter of 3 pixels. For example, (**A**) shows the raw P_x map and (**B**) the P_x after image processing. The corresponding fast Fourier transform (FFT) images of (**A**) and (**B**) were shown in (**C**) and (**D**), respectively.

SI References

1. Chen, L. Q. Phase-Field Method of Phase Transitions/Domain Structures in Ferroelectric Thin Films: A Review. *J. Am. Ceram. Soc.* **91**, 1835–1844 (2008).
2. Li, Y. L., Hu, S. Y., Liu, Z. K. & Chen, L. Q. Effect of electrical boundary conditions on ferroelectric domain structures in thin films. *Appl. Phys. Lett.* **81**, 427 (2002).
3. Li, Y. L., Hu, S. Y., Liu, Z. K. & Chen, L. Q. Effect of substrate constraint on the stability and evolution of ferroelectric domain structures in thin films. *Acta Mater.* **50**, 395–411 (2002).
4. Hong, Z. *et al.* Stability of Polar Vortex Lattice in Ferroelectric Superlattices. *Nano Lett.* **17**, 2246–2252 (2017).
5. Hong, Z. & Chen, L. Q. Blowing polar skyrmion bubbles in oxide superlattices. *Acta Mater.* **152**, 155–161 (2018).
6. Chen, L. Q. & Shen, J. Applications of semi-implicit Fourier-spectral method to phase field equations. *Comput. Phys. Commun.* **108**, 147–158 (1998).
7. Wang, J. J., Ma, X. Q., Li, Q., Britson, J. & Chen, L. Q. Phase transitions and domain structures of ferroelectric nanoparticles: Phase field model incorporating strong elastic and dielectric inhomogeneity. *Acta Mater.* **61**, 7591–7603 (2013).
8. Tagantsev, A. K. The role of the background dielectric susceptibility in uniaxial ferroelectrics. *https://doi.org/10.1080/00150198608008205* **69**, 321–323 (2011).
9. Zheng, Y. & Woo, C. H. Giant piezoelectric resistance in ferroelectric tunnel junctions. *Nanotechnology* **20**, 075401 (2009).
10. Das, S. *et al.* Observation of room-temperature polar skyrmions. *Nature* **568**, 368–372 (2019).
11. Padgett, E. *et al.* The Exit-Wave Power-Cepstrum Transform for Scanning Nanobeam Electron Diffraction: Robust Strain Mapping at Subnanometer Resolution and Subpicometer Precision. (2019).
12. Allen, L. J., D'Alfonso, A. J. & Findlay, S. D. Modelling the inelastic scattering of fast electrons. *Ultramicroscopy* **151**, 11–22 (2015).
13. Waasmaier, D. & Kirfel, A. New analytical scattering-factor functions for free atoms and ions. *Acta Crystallogr. Sect. A* **51**, 416–431 (1995).
